# Targeting NK Cells for Anticancer Immunotherapy: Clinical and Preclinical Approaches

**DOI:** 10.3389/fimmu.2016.00152

**Published:** 2016-04-21

**Authors:** Sebastian Carotta

**Affiliations:** ^1^Immune Modulation Department, Boehringer Ingelheim RCV, Vienna, Austria; ^2^The Walter and Eliza Hall Institute of Medical Research, Parkville, VIC, Australia

**Keywords:** natural killer cells, checkpoint inhibitors, immunotherapy, cancer, immune therapy

## Abstract

The recent success of checkpoint blockade has highlighted the potential of immunotherapy approaches for cancer treatment. Although the majority of approved immunotherapy drugs target T cell subsets, it is appreciated that other components of the immune system have important roles in tumor immune surveillance as well and thus represent promising additional targets for immunotherapy. Natural killer (NK) cells are the body’s first line of defense against infected or transformed cells, as they kill target cells in an antigen-independent manner. Although several studies have clearly demonstrated the active role of NK cells in cancer immune surveillance, only few clinically approved therapies currently exist that harness their potential. Our increased understanding of NK cell biology over the past few years has renewed the interest in NK cell-based anticancer therapies, which has lead to a steady increase of NK cell-based clinical and preclinical trials. Here, the role of NK cells in cancer immune surveillance is summarized, and several novel approaches to enhance NK cell cytotoxicity against cancer are discussed.

## Overview

Natural killer (NK) cells were identified in 1975 as a unique lymphocyte population that is clearly distinct from the other lymphoid lineages, such as T- and B-cells. NK cells were shown to differ from adaptive lymphocytes in respect to their morphology as well as in their capability to kill tumor cells without prior sensitization (1, 2). Since their discovery, research over the past 40 years significantly improved our understanding of the regulation of NK cells and has established several essential roles of NK cells during development in healthy individuals and during disease that can be therapeutically utilized.

Natural killer cells are the founding member of the innate lymphoid cell (ILC) family and are generally grouped based on their organ of development and tissue localization: we distinguish bone marrow-derived or adult conventional NK (cNK) cells, thymic-derived, fetal liver-derived, liver-resident, uterine-resident, and intestinal-resident NK cells. Adult cNK cells develop from the common lymphoid progenitor in the bone marrow in mice and humans and are considered the major NK cell subset responsible for tumor immune surveillance, albeit a role of the other subsets cannot completely be ruled out. During murine adult hematopoietic development, NK cell precursors are thought to be derived from a common innate lymphoid progenitor (CILP) and then mature through several progenitor stages into mature NK cells and migrate to several lymphoid and non-lymphoid tissues (3, 4). Peripheral NK cell maturation is then defined by the differential expression of CD11b, CD27, and KLRG1. Immature NK cells are defined as CD11b^−^CD27^+^KLRG1^−^ and mature NK cells as CD11b^+^CD27^+^KLRG1^−^ (M1) or CD11b^+^CD27^−^KLRG1^+^ (M2) (4–6). These different subsets differ in their ability to lyse target cells and their ability to secrete cytokines (5, 6). The mature CD11b^high^CD27^−^KLRG1^+^ NK cells are the dominant population in non-lymphoid organs except for the liver, where a distinct TNF-related apoptosis-inducing ligand (TRAIL)^+^CD49b^−^CD11b^low^ expressing population exists (7–9).

In contrast to murine NK cell development, the NK cell precursor populations in humans are currently not as well defined (10). Mature NK cells make up around 5–20% of peripheral blood lymphocytes. They are usually defined as CD3^−^CD56^+^ lymphoid cells and are subdivided into two major subpopulations, CD56^dim^CD16^+^ and CD56^bright^CD16^−^ cells. CD56^dim^ NK cells are the dominant subset in peripheral blood and spleen, express perforin, and are the most potent one in killing cancer cells (11–13). CD56^bright^ NK cells represent the main NK cell subset in lymph nodes and tonsils, lack perforin expression but are efficient producers of cytokines, such as IFN-γ, in response to the interleukins (IL)-12, IL-15, and IL-18. Thus, this subset is considered to be one of the key regulators of immune responses.

A major difference between NK and T cells is that NK cells can kill target cells instantly without needing prior sensitization, giving the adaptive immune reaction enough time to mount an antigen-specific immune response. Although the speed in which NK cells can kill infected or malignant cells is a big advantage when fast immune reactions are required, the ready-to-kill status of NK cells could be potentially dangerous for the body. Thus, NK cell activation is tightly regulated by activating the inhibitory receptors and the balance of the signaling through these receptors dictates if NK cells kill their target cells or remain inactive (14, 15). To prevent autoreactivity, NK cells express MHC class-I-specific receptors such as the killer cell immunoglobulin-like receptors (KIRs) in human, the lectin-like Ly49 dimers in the mouse, and the CD94–NKG2A heterodimers which exist on both, mice and humans. Binding of MHC-I molecules to these inhibitory receptors prevents cytolytic activity against healthy cells. During cancer progression, cancerous cells often decrease or even loose the expression of MHC-I on the surface, which allows them to evade T cell recognition and killing. However, loss of the MHC-I-mediated inhibitory signal on NK cells results in NK cell activation and cancer cell killing if no other inhibitory signals are active. In addition to the loss of inhibitory receptor signaling, NK cells can be directly activated by activating receptors, such as NKG2D, NKp30, NKp44, NKp46, 2B4, DNAM-1 (CD226), or CD16 (4, 6, 14, 16, 17). Although the ligands for some activating receptors have not yet been identified, it is currently believed that activating ligands are not expressed on healthy cells but are upregulated on diseased cells and that signaling through the activating receptors will dominate over the MHC-I-mediated inhibitory signaling. Besides the direct ligand–receptor interaction, NK cell functions are as well modulated by several cytokines. NK cells can be activated through type I interferons, IL-2, IL-12, IL-15, IL-18, and IL-21, whereas suppressive cytokines, such as transforming growth factor (TGF)-β or IL-10, can render NK cells inactive (18).

Several different pathways exist through which NK cells kill their target cells. On the one hand, NK cells induce apoptosis in their target cells by releasing lytic granules, such as granzyme B and perforin, *via* the formation of a lytic immunological synapse between the NK and target cells (19). Released perforin induces membrane perforation allowing the secretion of granzymes into the intracellular space inducing either caspase-dependent or -independent apoptosis. Another mechanism to kill is the induction of the death receptor-mediated apoptosis pathway. Here, FasL and TRAIL expressed on NK cells bind to Fas and TRAIL receptor triggering target cell apoptosis. In addition, NK cell-derived TNF-α can as well induce target cell apoptosis.

Despite the majority of current NK cell-mediated anticancer therapies focus on the lytic capability of NK cells, the indirect antitumor immunity capacity of NK cells should not be disregarded. NK cells are known to regulate the innate and adaptive immune response through the secretion of various cytokines, chemokines, adenosine, and growth factors (20, 21). NK cell-derived IFN-γ induces dendritic cell (DC) maturation leading to increased IL-12 production. IFN-γ as well induces the differentiation of CD8^+^ T cells into cytotoxic T cells (CTLs) and promotes the differentiation of CD4^+^ cells into Th1 T cells, which in turn promote the CTL response. NK cells not only enhance immune responses but also dampen T cell responses by either killing DC or inhibiting CD8^+^ T cell responses directly through IL-10 secretion. Our current understanding of the immune modulatory role of NK cells is, however, still limited and a better understanding will certainly open the door to novel NK cell-based immunotherapy approaches.

### Evidence for the Importance of NK Cells in Anticancer Immunosurveillance

An essential role for NK cells in human immune surveillance has been clearly established. Defects in human NK cell development or effector functions result in recurrent viral infections and in an increased risk of cancer development (22). Probably, the best evidence for the role of NK cells in anticancer immune surveillance comes from an epidemiological 11-year follow-up cohort study among a Japanese general population: the study demonstrated that high cytotoxic activity in peripheral blood lymphocytes is associated with reduced cancer risk, whereas low activity is associated with increased risk to develop various types of cancer (23). Subsequently, several other studies found that high levels of tumor infiltrating NK cells are associated with favorable outcome in patients with colorectal carcinoma, gastric cancer, and squamous cell lung cancer (24). Indicative of an important role of NK cells in tumor control, cancer cells have developed several strategies to escape from NK cell recognition. Tumor cells can upregulate ligands for inhibitory receptors or secrete immune suppressive factors, including TGF-β, IL-10, prostaglandin E2, indoleamine 2,3-dioxygenase (Ido), and adenosine (25–29). Shedding of ligands for activating receptors represents another potential strategy by tumor cells to reduce the amount of activating ligands on the surface of tumor cells and/or induce NK cell desensitization (30–33). However, a recent report questioned the shedding mechanism as a way to invade the immune surveillance. In the mouse model, Deng et al. demonstrated that a shed form of the mouse NKG2D ligand MULT1 can lead to boosting of NK cell activity (34).

Despite ample evidence that NK cells participate in the fight against cancerous cells, very few therapeutical approaches currently exist that are targeting NK cells. However, support for the potential of NK cells as therapeutic targets is coming from approved cancer cell-targeting therapies as several drugs have been recently demonstrated to additionally modulate NK cell activity. In the next section, I will review the effect of a few of such therapies.

### Cancer Cell-Targeting Drugs with NK Cell-Modulating Activity

Noteworthy, many targets of current cancer therapies are expressed in cancer cells and immune cells. It is therefore not surprising that few cancer therapies not only impact on cancer cell survival and proliferation but also influence the immune system. But because the majority of cancer-targeting drugs is generally tested preclinically for their efficacy and safety in xenograft models that lack a functional immune system, this effect is often not apparent. Indeed, recent studies have shown that radiotherapy or chemotherapies, such as Ara-C, cisplantin, or 5-FU, can lead to increased expression of NK cell activating ligands and thus enhance NK cell recognition and killing (35). More recently, several precision medicine drugs have additionally been demonstrated to increase NK cell-mediated tumor killing (36, 37). The proteasome inhibitor bortezomib, currently successfully used in the treatment of multiple myeloma, can induce the expression of ligands of NK cell activating receptors. Another example is the immunomodulatory (IMiD) drug lenalidomide, which is approved for the treatment of multiple myeloma and myelodysplastic syndromes (MDS). Besides having a direct effect on cancer cells and angiogenesis, lenalidomide modulates the immune response by increasing the NK cell number in the periphery. The exact mode of action of lenalidomide on NK cells is currently not clear. Several modes of actions have been proposed. Lenalidomide might increase NK cell activation indirectly by upregulating ligands on tumor cells and induce the expression of NK cell stimulatory cytokines such as T cell-derived IL-2 or directly by lowering the threshold for NK cell activation (38, 39). A better understanding of the mode of actions of lenalidomide on NK cells will be certainly crucial to design rational combination therapies. This is highlighted by the fact that lenalidomide in combination with the anti-CD20 antibody rituximab can lead to increased efficacy in B cell malignancies by enhancing the antibody-dependent cell-mediated cytotoxicity (ADCC) effect, but the combination with dexamethasone inhibits the immune-stimulatory effect of lenalidomide on NK cells, potentially *via* suppressing IL-2 production in CD4^+^ T cells (40–42).

However, cancer-targeting drugs not always enhance the activity of immune cells, but in some cases, have been reported to exert detrimental effects on the immune system. Ibrutinib is a novel irreversible inhibitor of Bruton’s tyrosine kinase that shows promising effects in the treatment of mantle cell lymphoma (MCL) and chronic lymphocytic leukemia (CLL). Rituximab in combination with chemotherapy is currently standard of care in CD20^+^ B-cell malignancies and thus a potential combination of ibrutinib with rituximab is attractive. However, recent studies demonstrated that ibrutinib actually antagonizes the ADCC effect of rituximab in CD20^+^ B-cell lymphoma due to Ibrutinib irreversible binding to IL-2 inducible tyrosine kinase (ITK), which is required for FcR-stimulated NK cell function (43, 44).

Another example is ruxolitinib, a small molecule inhibitor of the JAK 1/2/3 signaling pathway. Ruxolitinib is currently approved for the treatment of myelofibrosis (MPN). As several cytokines regulate NK cell development and function *via* the JAK/STAT signaling pathway, patients who were treated with ruxolitinib had drastically reduced circulating NK cell numbers. *In vitro* studies further demonstrated that ruxolitinib potently inhibited the cytokine-induced cytolytic activity of NK cells (45). However, importantly, NK cell depletion by ruxolitinib was reversible as the NK cell levels rose back to normal values in patients who stopped ruxolitinib treatment. Thus, when combined with NK cell-based immunotherapies, proper scheduling of therapeutic drugs will be crucial.

### Clinical or Preclinical Therapies Augmenting NK Cell Function

#### Checkpoint Inhibitors

##### PD-1

**Figure 1:**
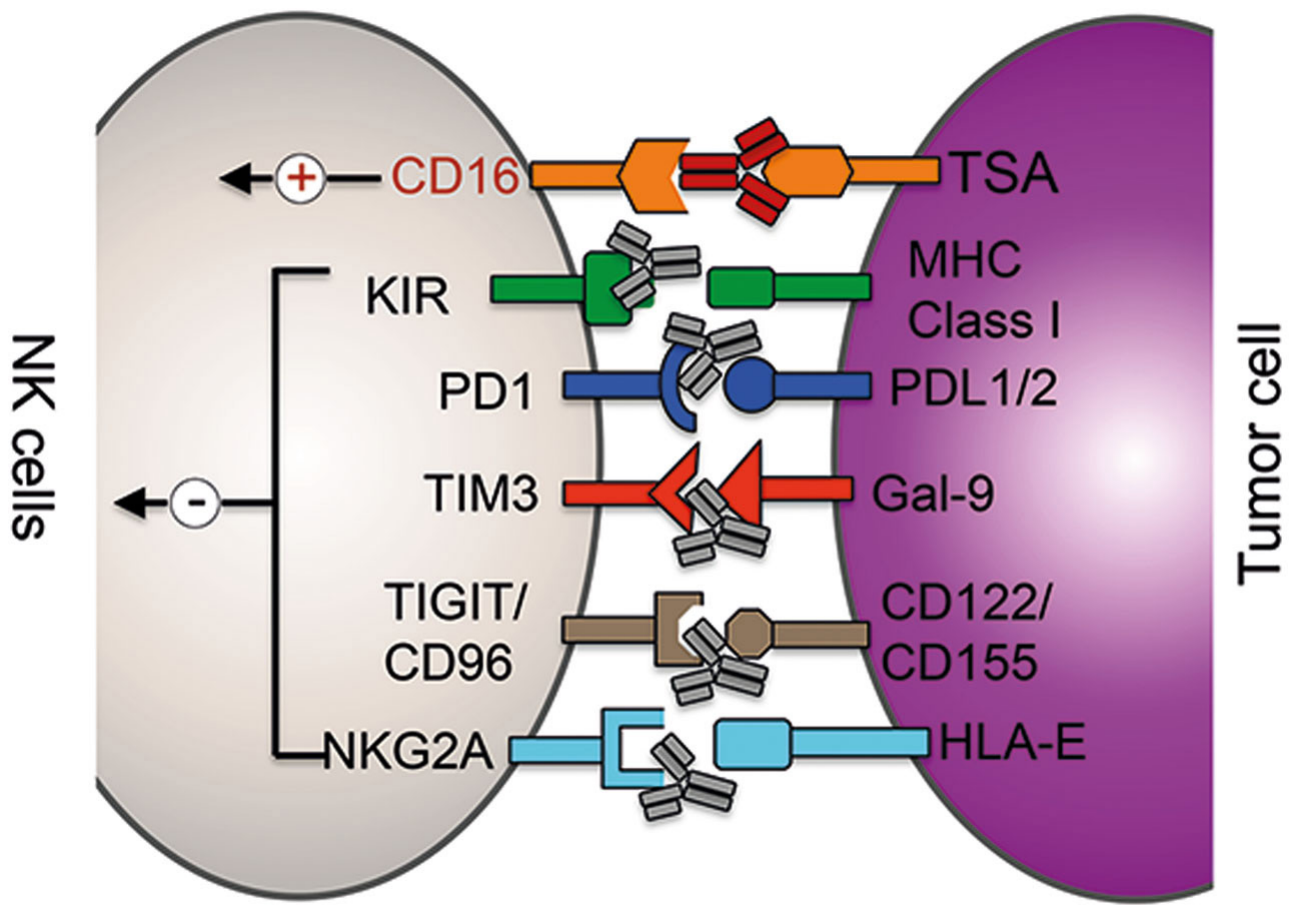
**Clinical and preclinical therapies augmenting NK cells function**. mAb (gray)-mediated blockade of the interaction between inhibitor receptors (in black) expressed on NK cells with the respective ligands on tumor cells (or suppressor cells) results in increased cytolytic potential of NK cells. ADCC therapy: binding of mAbs (red) to tumor-specific antigens (TSA) results in the activation of NK cells *via* the activation of the activating receptor CD16 (red).

Checkpoint inhibitors are currently the most promising approaches among immunotherapies. Treatment with anti-CTLA4 or anti-PD-1 antibodies restores T cell activity in cancer patients and has resulted in durable tumor regression in some patients. And the combination of both checkpoint inhibitors was able to further enhance the therapeutic benefit significantly (46, 47). The expression of PD-1, however, is not restricted to activated and exhausted T cells but can be detected on subsets of other immune cells and even on melanoma cells (48–51). A recent report demonstrated that NK cells from multiple myeloma and renal carcinoma patients expressed PD-1 on their surface and engagement of PD-1 signaling reduced their cytolytic potential (Figure 1) (50, 51). Treatment of patient-derived PD-1^+^ NK cells with an anti-PD-1 antibody (pidilizumab, CT-011) was able to increase NK cell-mediated killing of autologous cancer cells *in vitro* (50). A recent phase II trial tested the efficacy of pidilizumab with rituximab in patients with relapsed follicular lymphoma and found that the combination is well tolerated and indicated favorable therapeutic effects when compared to rituximab single treatment (52). The therapeutic benefit of re-invigorating PD-1^+^ NK cells in cancer patients is currently not well understood, and the major therapeutical effect is certainly due to re-activation of exhausted T cells. However, a contribution of NK cells to the observed therapeutic benefit cannot be excluded, especially in hematological malignancies, and thus warrants further investigation.

##### TIM-3

TIM-3, also known as HAVCR2, is another immune checkpoint receptor that is currently being tested in preclinical models for its potential to re-invigorate exhausted T cells in cancer patients (53). Resting T cells express low levels of TIM-3, and its expression is strongly upregulated in activated and exhausted T cells. Antibody-mediated blockade of TIM-3 signaling was able to reverse the exhausted phenotype of CD4^+^ and CD8^+^ T cells in melanoma patients proving the inhibitory function of TIM-3 in T cells (54). Human tumor-derived CD8^+^ T cells often coexpress TIM-3 and PD-1, and preclinical studies in several murine tumor models demonstrated that the combination of TIM-3 and PD1 blocking antibodies can significantly increase the reversal of T-cell exhaustion. Like PD-1, TIM-3 expression is not restricted to T cells but can also be detected on murine and human NK cells (55, 56). In contrast to T cells, where TIM-3 surface expression marks dysfunctional T cells, TIM-3 is expressed on virtually all human NK cells and is further upregulated on cytokine-activated NK cells (Figure 1). Thus, TIM-3 expression is regarded as a marker for mature NK cells. Currently, the functional role of TIM-3 on NK cells is highly controversial. Ndhlovu et al. recently demonstrated that crosslinking of TIM-3 *via* anti-TIM-3 antibodies on the human NK cell line NKL or on human PBMC-derived NK cells significantly decreased their cytolytic ability (57). In stark contrast to these findings, Gleason et al. showed that activation of TIM-3 through the ligand Gal-9 actually increased the production of IFN-γ in NK cells (58). A more recent report suggested that the discrepancy between these two studies might origin from the different experimental layout as well as by the fact that NK cell lines and NK cells from healthy donors have been analyzed (56). Therefore, they tested the effect of TIM-3 blockade on NK cells derived from advanced melanoma patients. da Silva et al. found that TIM-3 surface expression increases with the progression of the cancer, TIM-3^+^ NK cells display an exhausted phenotype and that high expression levels correlated with poor prognosis. More importantly, when TIM-3^+^ NK cells derived from melanoma patients were incubated with anti-TIM-3-coated beads, TIM-3 activation resulted in modest, but statistically significant decrease in IFN-γ secretion and degranulation. In summary, the function of TIM-3 on NK cells is currently controversial, and more detailed studies on the role of TIM-3 on NK cells derived from cancer patients are required to fully understand the role and therapeutic potential of TIM-3 blockade in NK cell therapy.

##### NKG2A

The heterodimer CD94/NKG2A is another checkpoint inhibitor complex whose expression is shared between T and NK cells (Figure 1). Human CD94–NKG2A/C/E heterodimers recognize the non-classical MHC class-I molecule HLA-E in humans and Qa-1 in mice, which is expressed on many lymphoid cells (59). The NKG2A chain of the CD94/NKG2A receptor contains two immunoreceptor Tyr-based inhibitory motifs (ITIMs) in its cytoplasmic tail and HLA-E/NKG2A interaction results in a dominant inhibitory signaling event that causes a strong decrease in NK cell effector functions. Several solid cancer and hematological malignancies use the upregulation of HLA-E expression as an immune escape mechanism in order to evade killing by NK cells and T cells (60, 61). Therefore, the use of a blocking NKG2A antibody could be another useful addition to the steadily growing list of T/NK cell-targeting immunotherapy approaches. Monalizumab (previously IPH2201) represents such an anti-NKG2A checkpoint inhibitor and is currently under clinical investigation. In a phase I/II trial, monalizumab is currently being evaluated in head and neck cancer and ovarian cancer. Furthermore, the effects of the combination of monalizumab with ibrutinib (CLL, phase I/II), cetuximab (head and neck, phase I/II), and duvalumab (solid tumors, phase I/II) are currently investigated.

##### TIGIT and CD96

TIGIT, CD96, and CD226 (DNAM-1) belong to the same immunoglobulin family of receptors that interact with nectin and nectin-like proteins (16). Although all three receptors are expressed on NK cells and can bind CD155 and CD112, ligand binding is triggering different responses (Figure 1). CD226 is an activating receptor that is important for NK cell-mediated tumor surveillance and ligand binding increases the cytotoxic potential of NK cells against target cells (16). On the other hand, TIGIT and CD96 contain ITIM motifs in their cytoplasmic domains and are inhibitory receptors. Although CD155 present on tumor cells can induce CD226-dependent immunosurveillance, the expression of CD96 and TIGIT on the same cell can counterbalance CD226 activity. While activation of TIGIT on human NK cells inhibited *in vitro* cell killing of target cells, antibody-mediated blocking of TIGIT significantly increased the cytolytic activity (62). Using CD96^−/−^ mice, Chan et al. recently demonstrated that loss of CD96 expression resulted in improved tumor control of methylcholanthrene (MCA)-induced fibrosarcoma and lung metastasis (63). Although blockade of TIGIT *in vitro* increased the cytolytic activity of NK cells, the improved antitumor response in *CD96*-deficient mice was dependent on IFN-γ production by NK cells. It is currently not clear why NK cells express simultaneously two inhibitory receptors on the same cell to counteract CD226 activation, but data from the Smyth group indicate that the two receptors control different NK cell effector functions: TIGIT may predominantly inhibit the cytolytic potential of NK cells, whereas CD96 regulates the production of IFN-γ (16, 63). Future research will unravel which of these two receptors should be inhibited to increase tumor surveillance in human patients or if inhibition of both receptors simultaneously will result in improved NK/T cell-mediated cancer cell control.

##### Killer Cell Immunoglobulin-Like Receptors

Natural killer cells express inhibitory KIRs that recognize self-MHC class-I molecules to prevent cytotoxicity against host cells (Figure 1). As tumor cells express the same MHC class-I molecules than healthy tissue, the interaction between self-HLA on cancer cells with KIRs on NK cells reduces the cytolytic activity of NK cells against tumor cells (64–68). Therefore, KIRs represent an interesting class of targets for NK cell-specific checkpoint inhibition. Following this reasoning, a humanized KIR-blocking monoclonal antibody (mAb), IPH2101, has been generated and is currently tested in clinical trials. IPH2101 is specific against three inhibitory KIRs, namely, KIR2DL-1, -2, and -3, that are specific for all HLA-C molecules. Preclinical *in vitro* and *in vivo* studies demonstrated that IPH2101-mediated blockade of KIRs on human NK cells significantly increased cytolytic activity against tumor cells (69–71). Importantly, no sign of autoimmunity was observed in treated mice. Confirming the results of the preclinical studies, no severe side effects were observed in clinical phase I and phase II trials in patients with acute lymphoblastic leukemia or multiple myeloma (72–74). Although the current clinical trials using IPH2101 as a monotherapy did not demonstrate significant antitumor efficacy, based on the encouraging preclinical data of IPH2101 and the recent success of combining checkpoint inhibitors, there is still hope that the rational combination with other drugs can lead to improved clinical antitumor responses. Potential combination partners could be the above described checkpoint inhibitors and other IMiD drugs, such as lenalidomide or NK cell activating cytokines.

##### Redirection of NK Cell Cytotoxicity *via* Biologics

Antibodies recognizing tumor-specific epitopes represent a highly efficient strategy to direct the cytolytic activity of NK cells against malignant cells. One approach that is currently successfully used in the clinics is *ADCC-based therapies*. NK cells express the activating surface receptor CD16 (FcγRIIIA), which specifically binds the constant region (Fc) of immunoglobulin G (IgG) antibodies. The interaction between CD16 on NK cells and the Fc portion of a tumor-specific IgG antibody bound on cancer cells results in the activation of NK cells and subsequently killing of respective tumor cells (Figure 1). Currently, several ADCC therapies are tested in clinical trials or are already successfully used in the clinics, such as α-CD20, α-GD2, α-Her2, and α-EGFR mAbs. The current status of ADCC therapies were summarized in recent review (75). However, it is important to mention that CD16 is expressed not only on NK cells but also on activated myeloid subsets. Therefore, several hematopoietic lineages are likely to contribute to the observed therapeutic effects of ADCC (75). Besides mAbs, bispecific or trispecific killer engagers (BiKEs and TriKEs) are currently developed. These antibodies are able to target either one (BiKE) or two (TriKE) different antigens on the tumor cell and bind to another epitope of the CD16 receptor leading to improved NK cell-mediated ADCC effect [for a review on BiKEs and TriKEs, please see Wang et al. (75) and Kontermann and Brinkmann (76)].

#### Targeting Immune Suppressive Signaling

##### Transforming Growth Factor-β

**Figure 2:**
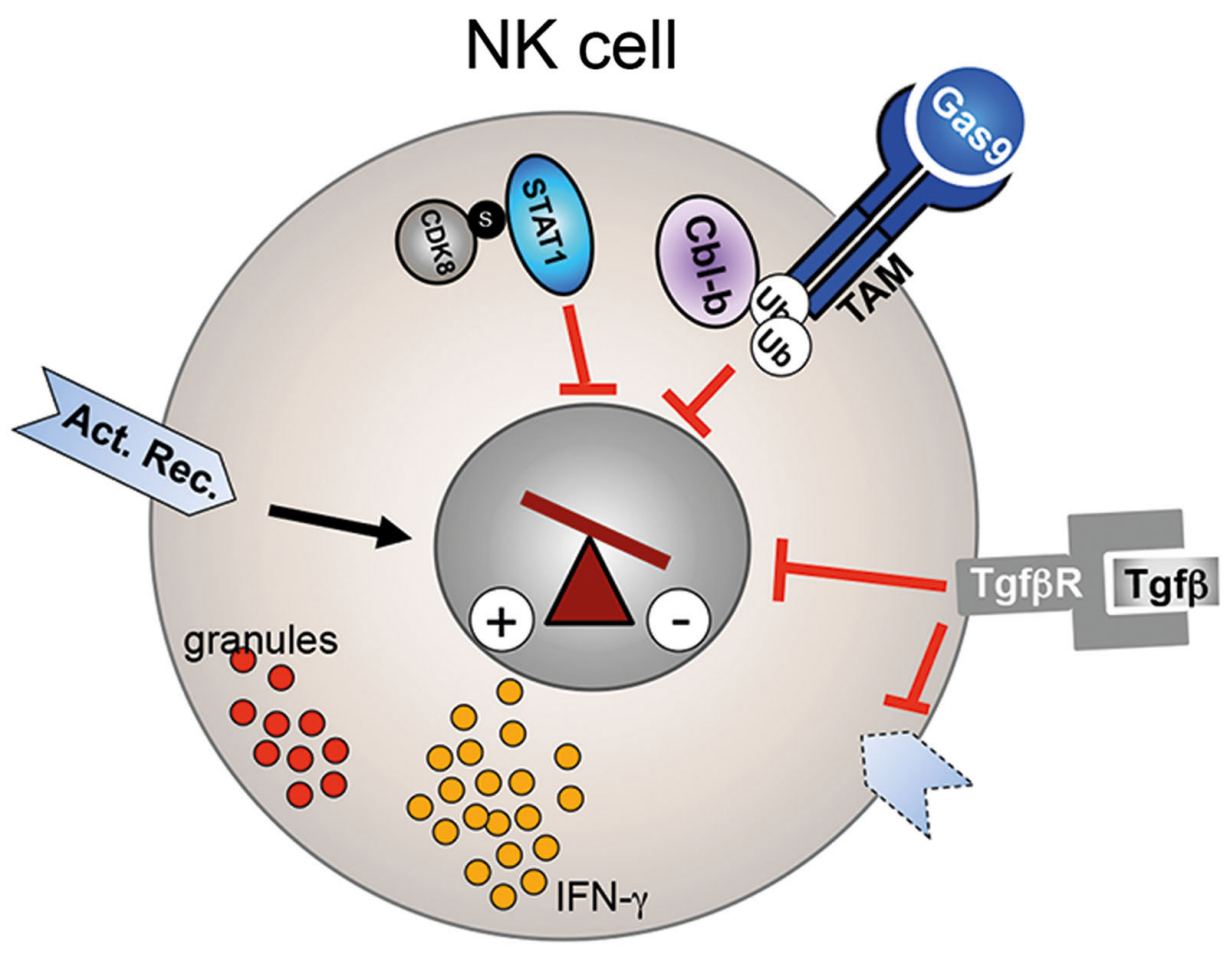
**Regulation of NK cell activity**. CDK8-mediated STAT1–Ser727 phosphorylation inhibits the cytolytic activity of NK cells. Similarly, Cbl-b-mediated ubiquitinylation of the TAM receptor results in reduced perforin, granzyme B (red granules), and IFN-γ secretion. TGF-β signaling reduces as well NK cell effector function and leads to downregulation of activating receptors (dotted receptor).

Secretion of TGF-β by tumor cells or the tumor microenvironment has copious effects on tumor progression and on the immune system (77). During cancer progression, TGF-β can play a key role in tumor immune escape. TGF-β levels are often increased in the serum of cancer patients and elevated levels correlate with systemic inhibition of the immune system and poor prognosis (78, 79). Like CD8^+^ T cells, NK cells from patients with elevated TGF-β levels displayed reduced cytotoxicity and had reduced expression levels of the activation markers, NKG2D, NKp46, or increased expression of NKG2A (Figure 2) (28, 80). *Ex vivo* treatment of patient-derived NK cells with neutralizing anti-TGF-β mAbs was able to restore activating receptor expression, proliferation, and cytokine secretion (29). Coculture of healthy human NK cells with human ALL blasts reduced their cytolytic activity and IFN-γ production. This effect was mediated by ALL-derived TGF-β as an anti-TGF-β blocking antibody was able to rescue NK cell functions (28). In line with a direct effect of TGF-β signaling on NK cell receptor expression and NK cell function, *in vitro* incubation of human NK cells with TGF-β resulted in downregulation of NKp30 and NKG2D, inhibition of IL-15 induced NK cell proliferation and IFN-γ secretion (81). Therefore, targeting TGF-β signaling in NK cells represents an attractive immunotherapy approach in cancer patients with elevated TGF-β levels. However, due to the many functions of TGF-β in normal tissue, cancer cells, tumor microenvironment, and immune cells, developing potent inhibitors with a low toxicity profile is challenging (82, 83). Currently, several approaches to inhibit TGF-β signaling are pursued to increase efficacy and limit toxicity in preclinical and in clinical trials with various successes. Approaches include ligand traps, antisense oligonucleotides, receptor kinase inhibitors, and peptide aptamers (84). In summary, while it is currently to early to judge if anti-TGF-β immunotherapies will become reality, preclinical studies yielded enough convincing results that interference with the TGF-β pathway is able to increase NK cell (and T cell) effector functions to warrant further new drug development.

## Exploratory Targets – The Next Wave?

### Intracellular Targets

The majority of approved immunotherapies target surface receptors on immune cells *via* mAbs that either inhibit protein–protein interactions between immune cells and other cell types (antagonistic antibodies) or activate target receptor on certain immune cells (agonistic antibodies). Whereas in cancer-targeting therapeutic approaches small molecule drugs dominate, this class of therapies is conspicuously missing or at least under-represented in current anticancer immunotherapy approaches. Targeting intracellular proteins *via* small molecules significantly extends the pool of potential novel immunotherapy targets. Different inhibitory receptors often use the same intracellular pathways to relay their inhibitory signal into the nucleus. Thus, by inhibiting such pathways, it might be possible to therapeutically affect several inhibitory receptors at the same time *via* inhibiting one molecule. The phosphatases Src homology region 2 domain-containing phosphatase (SHP)-1 (PTPN6) and SHP-2 (PTPN11) are two good examples as several inhibitory receptors on T cells and NK cells have been shown to recruit SHP-1 and/or SHP-2 after activation (85). Of course, targeting intracellular pathways with such broad activity can come with a cost, in this case, the potential increase of toxicity. Other advantages of small molecules over biologicals are excellently summarized in a recent review (86). In this next section, I will discuss a few recently published molecules that play a role in the regulation of NK cell function and might represent potential future targets for NK cell-mediated immunotherapy.

#### Casitas B-Lineage Lymphoma Proto-Oncogene-b

Post-transcriptional modification of proteins, such as ubiquitination, is an important regulatory mechanism for the fine-tuning of several pathways. Recent studies demonstrated that the E3 ligase Casitas B-lineage lymphoma proto-oncogene-b (Cbl-b) is a key regulator of the immune response against cancer (87). Cbl-b is highly expressed in most murine and human immune cells, including T and NK cells. The importance of Cbl-b in antitumor immune response was discovered when *Cbl-b*-deficient mice spontaneously rejected several different tumors (88, 89). Tumor rejection was first considered to be mainly mediated by CD8^+^ T cells. However, a recent study elegantly demonstrated that Cbl-b also plays a key role in NK cell-mediated tumor control. Deletion or pharmacological inhibition of Cbl-b increased the cytolytic potential, proliferative capacity, and IFN-γ secretion of NK cells *in vitro* (Figure 2) (90). More importantly, tumor growth and metastasis were significantly decreased in Rag2^−/−^Cbl-b^−/−^ mice when compared to Rag2^−/−^Cbl-b^wt^ mice. Antibody-mediated NK cell depletion and inactivation *via* anti-NK1.1 and anti-NKG2D antibodies, respectively, abrogated this antitumor response, identifying the NK cell lineage as the main mediator of the observed antitumor effect in these mice. Furthermore, NK cell activity was completely dependent on the catalytic domain of the E3 ligase of Cbl-b. Through an *in vitro* ubiquitinylation screen of 9000 human proteins, the authors then identified the TAM receptor tyrosine kinases AXL, TYRO3, and MER as targets of Cbl-b. Indeed, activation of TAM receptors on wild-type NK cells *via* the natural ligand Gas6 suppressed IFN-γ secretion *in vitro*, whereas *Cbl-b*-deficient NK cells were resistant to this inhibition. These date therefore indicate that TAM or Cbl-b is potentially suitable targets for NK cell-mediated immunotherapy. Indeed, Paolino et al. developed a highly selective TAM kinase inhibitor, LDC1267, which increased the lytic activity of NK cell against B16F10 melanoma cells *in vitro* and *in vivo* in an adoptive transfer mouse model. Furthermore, intra-peritoneal injection of LDC1267 resulted in a decrease of micro-metastases in mice that were injected with the syngenic tumor cell line 4T1. In summary, interference of TAM receptor activity represents an interesting novel immunotherapy approach. Alternatively, a small molecule inhibitor of Cbl-b potentially could increase NK cell and T cell effector function against cancer cells (87). It is currently unclear how toxic such a small molecule would be as Cbl-b is expressed in many hematopoietic cell types. However, *Cbl-b*-deficient mice are viable and do not show signs of severe autoimmunity, thus a therapeutic window might exist.

#### CDK8

Natural killer cell development and functions are tightly regulated by several cytokines, such as IL-2, IL-12, IL-15, or type I interferons. The JAK/STAT pathway is playing a central role in relaying the effect of these different cytokines into the nucleus. IL-2 and IL-15 promote NK cell development and homeostasis mainly *via* activation of the transcription factor signal transducer and activator of transcription protein 5 (STAT5) (91, 92). On the other hand, STAT1 plays an essential role on the regulation of NK cell effector function (93). Type I interferon and IL12 induce STAT1 activation, resulting in increased cytotoxicity and IFN-γ secretion. Not surprisingly, therefore, deletion of STAT1 results in impaired NK cytolytic activity *in vitro* and reduced tumor rejection *in vivo*, despite normal numbers of NK cells, whereas *STAT5*-deficient mice lack NK cells completely (94–96). The activity of STAT1 is mainly regulated post-transcriptionally. For activation and translocation of STAT1 into the nucleus, STAT1 has to be phosphorylated at tyrosine 701 (Y701) by the Janus kinase JAK. A recent report demonstrated a role of STAT1–Ser727 phosphorylation in the regulation of the lytic potential of NK cells (96). Resting NK cells showed a basal level of STAT1–Ser727 phosphorylation, which increased after *in vitro* stimulation with either IFN-β or IL-12. Interestingly, in contrast to Y701 phosphorylation, Ser727 phosphorylation resulted in an inhibitory effect on NK cell activity, indicating that phosphorylation of STAT1–Ser727 represents a negative feedback in activated NK cells loop to prevent over-stimulation. *Ex vivo* isolated STAT1–Ser727A mutant NK cells had increased lytic potential against a range of tumor cell lines *in vitro* and secreted increased levels of granzyme B and perforin. *In vivo*, STAT1–Ser727A mutant mice showed increased anticancer immunosurveillance against the murine tumor lines B16F10, 4T1, and a v-abl transformed leukemic cell line. Through the generation of *Rag1*^−/−^ *STAT1–Ser727A* mice that lack B and T cells but have NK cells, Putz et al. demonstrated that the antitumor effect was strictly dependent on NK cell activity. Interestingly, By contrast, the molecules mentioned above which limited both, the production of lytic granules and IFN-γ secretion, STAT–1Ser727 repressed perforin and granzyme B but induced IFN-γ secretion slightly. The authors then identified the cyclin-dependent kinase 8 (CDK8) as the kinase responsible for the phosphorylation of STAT1 as Ser727 (Figure 2). Knock-down of CDK8 reduced STAT-1–Ser727 phosphorylation and slightly increased target cell lysis in an *in vitro* killing assay. Thus, pharmacological inhibition of CDK8 kinase activity might represent an attractive approach to augment NK cell-mediated anticancer immunosurveillance. Currently, the open questions are if the same effect will be seen in human NK cells and how toxic a CDK8 inhibitor will be given the broad expression of CDK8. However, CDK8 therapy could potentially have another positive anticancer effect: CDK8 has been previously shown to be an oncogenic driver in colorectal cancer, breast cancer, and melanoma (97–99). Therefore, one could envision that CDK8 inhibitors, on the one hand, induce tumor cell death and, on the other hand, stimulate NK cell activity.

#### EZH2

The H3K27 methyltransferase enhancer of zeste homolog 2 (EZH2) is essential for many biological processes, including the regulation of immune responses, and is overexpressed in several cancers. Therefore, the pharmacological targeting of EZH2 is an interesting approach for future immunotherapies (100, 101). A recent study demonstrated a role of EZH2 in NK cell development (102). Absence of EZH2 in human and murine hematopoietic progenitors resulted in an increased commitment to the NK cell lineage. In addition, *EZH2*^−/−^ NK cells expressed higher levels of NKG2D, IL2Rα, IL7Rα, and the lytic proteases granzyme A and B. The negative regulation of NK cell development and function by EZH2 was dependent on its methyltransferase activity as pharmacological inhibition of EZH2 resulted in a similar phenotype when compared to *EZH2*^−/−^ NK cells. These data suggest that EZH2 inhibitors may not only have an effect on cancer cell growth and survival but potentially can augment NK cell number and function in patients. However, this remains to be tested as the above-mentioned study mainly focused on the effect of EZH2 on *in vitro* NK cell differentiation, and little data are currently available on the effect on mature NK cells. Nevertheless, several studies are testing the efficacy of adoptive transfer of *ex vivo* expanded NK cells as an immunotherapy approach. Thus, it will be of interest if the inhibition of EZH2 during the NK cell differentiation/expansion phase can lead to an increase in cell number and augment the activity of NK cells.

## Conclusion

Although the ability of NK cells to kill malignant cells efficiently has been demonstrated several decades ago, the potential of NK cell-based immunotherapy is often questioned due to modest clinical responses of current therapies. Recent advances in our understanding of NK cell biology yielded already in promising new therapeutic approaches and continuous investigation of the mechanisms that regulate NK cell function will result in improved and more efficacious therapies in the future.

Many of the above described targets are not specific to NK cells, but often also function in other therapeutically interesting immune cells, such as T cells. Although the close relationship between NK and T cells makes if often difficult to identify how much of the therapeutic effect is due to NK cell activity, therapies that activate both effector cells are highly interesting as they are able to combine the therapeutic effects of both cell types (103). Until recently, only few experimental approaches existed to test the potential of NK cells in antitumor therapy. The most common and most feasible approach represented the antibody-mediated depletion of NK cells to investigate tumor growth in the presence or absence of NK cells. However, due to the lack of specific NK cell markers that can be targeted for depletion, it is often unclear if other cell types, such as T cells, have been affected as well. Recently, a novel NK cell-less mouse model has been established *via* the conditionally deletion of Mcl1 in NK cells (Mcl1^fl/fl^ NCR1^Cre^) (104). As MCL1 expression is essential for NK cell survival, virtually no residual NK cell subsets in all anatomical locations tested have been detected. As NCR1 is expressed as well on a subset of ILC3 cells in the gut, this mouse models lacked all NK cells and NCR1^+^ ILC3 cells. Nevertheless, this genetically engineered mouse represents an attractive model to test the specific role of NK cells in various disease settings.

Natural killer cell therapy as a monotherapy is unlikely to be curative for most, if not all, cancer types and a critical parameter for successful NK cell therapies will be the choice of combination partners. Therefore, future studies that investigate the interaction of NK cells with the other components of the immune system will be crucial for the optimal design of combination therapies.

In summary, while the potential of NK cell therapy is currently still not entirely clear, the recent advances in our understanding of NK cells certainly have resulted in novel, promising approaches, and it is very likely that future discoveries will continue to improve the efficacy of NK cell-based therapies.

## Author Contributions

The author confirms being the sole contributor of this work and approved it for publication.

## Conflict of Interest Statement

The author is an employee of Boehringer Ingelheim.
